# Influence of venous emptying on the reactive hyperemic blood flow response

**DOI:** 10.1186/1476-5918-6-3

**Published:** 2007-03-14

**Authors:** Zeki Bahadir, Eric Tisdell, Arturo A Arce Esquivel, Devon A Dobrosielski, Michael A Welsch

**Affiliations:** 1 Department of Kinesiology, Louisiana State University, Baton Rouge, LA, USA

## Abstract

**Background:**

Previous research indicates that venous emptying serves as a stimulus for vasodilation in the human forearm. This suggests the importance of recognizing the potential influence of venous volume on reactive hyperemic blood flow (RHBF) following occlusion. The purpose of this study was to examine the influence of venous emptying on forearm vascular function.

**Methods:**

Forearm RHBF, venous capacitance and venous outflow were examined in 35 individuals (age = 22 ± 2 years), using mercury in-Silastic strain gauge plethysmography, at rest and following five minutes of upper arm occlusion using standard procedures (Control). In addition, the same measures were obtained following five minutes of upper arm occlusion preceded by two minutes of passive arm elevation (Pre-elevation).

**Results:**

Average resting arterial inflow was 2.42 ± 1.11 ml·100 ml^-1^·min^-1^. RHBF and venous capacitance were significantly greater during Pre-elevation compared to Control (RHBF; Pre-elevation: 23.76 ± 5.95 ml·100 ml^-1 ^·min^-1 ^vs. Control: 19.33 ± 4.50; p = 0.001), (venous capacitance; Pre-elevation: 2.74 ± 0.89 % vs. Control: 2.19 ± 0.97, p = 0.001). Venous outflow did not differ between the two conditions.

**Conclusion:**

Venous emptying prior to upper arm occlusion results in a significant greater RHBF response and venous capacitance. Recognition of the influence of venous volume on RHBF is particularly important in studies focusing on arterial inflow, and also provides further evidence for the interplay between the venous and arterial system.

## Background

Venous occlusion plethysmography is a popular tool to study limb blood flow in healthy humans and those with disease [1]. The basis of the technique is that a "collecting" cuff is inflated around the upper or lower limb to a pressure less than diastolic so that arterial inflow may continue whereas venous outflow is obstructed. Under this condition, the limb "swells" and the volume increases [1]. Venous occlusion plethysmography allows one to examine the vasodilator response to reactive hyperemia. Both metabolic and myogenic influences appear to play a major role in the reactive hyperemic response.

In general, younger [2], fitter [2,3], and healthier [4] individuals exhibit greater reactive hyperemic blood flow (RHBF) responses following a period of occlusion. Moreover, reactive hyperemic responses are sensitive to interventions such as exercise training [2,3], diet [5], and pharmacology [6,7]. Recently, Tschakovsky and Hughson [8] showed that venous emptying served as a powerful stimulus for a transient vasodilation of the brachial artery, which substantially elevated arterial inflow. However, these authors did not examine the influence of the emptying of the veins in the limb under study on the reactive hyperemic response following a period of occlusion. Such information is important, as the venous volume appears to have a direct impact on the rate of arterial inflow. Moreover, if reactive hyperemia is a dependent measure in interventional trials it may be important to account for the venous volume as these volumes may themselves change throughout an intervention.

Therefore, the aim of this research study was to examine the influence of arm elevation on the RHBF response. An additional aim was to examine the influence of arm elevation on measures of venous function.

## Methods

### Study Participants

A total of 35 individuals from the host institution were recruited to participate in this study. Participants were free of any overt symptoms of disease. Individuals at high risk for adverse responses to exercise and/or taking any medication that may affect cardiovascular function were excluded from participation. Smokers and individuals with acute medical conditions (e.g. orthopedic injury), active infection and/or on pharmacotherapy with known vascular effects (e.g. anti-inflammatory therapy, cardiovascular medications) were also excluded. Following a comprehensive explanation of the proposed study, its benefits, inherent risks and expected commitments with regard to time, all participants signed an informed consent approved by the IRB of the host institution.

### Experimental Design

An observational prospective study was designed to examine the influence of venous emptying on forearm vascular function. Each participant was examined during one visit at which time blood flow measurements were obtained under two different conditions.

### Vascular Function Assessments

Participants were instructed to refrain from food, alcohol or caffeine for 12 hours, and rigorous physical activity for 24 hours before the procedures. Upon arrival to the laboratory participants were placed in a supine position for 10 minutes prior to evaluation of vascular function. During this time, blood pressure cuffs were positioned around the non-dominant upper arm and wrist of the participants, and a mercury-in-silastic strain gauge placed around the forearm approximately 10 cm distal to the olecranon process [2]. The strain gauge was then connected to a plethysmograph (EC-5R system, Hokanson; Bellevue,WA). The forearm was extended, slightly supinated and supported by a styrofoam block ensuring the arm was above heart level.

Vascular function indices, including arterial inflow, venous capacitance and outflow, and RHBF were then obtained at rest and following five minutes of forearm occlusion. Prior to each measurement, hand circulation was occluded for one minute by inflating a wrist cuff to 240 mmHg. All blood flow measures were reported in ml·100 ml^-1^·min^-1^. Prior to, during and following each procedure, blood pressure was obtained in the contralateral arm and heart rate was recorded using ECG electrodes. The reproducibility of these procedures in our laboratory has been previously reported [3].

#### Rest

Forearm blood inflow and outflow were recorded as follows: (1) hand circulation was occluded by inflating the cuff at the wrist to 240 mmHg; (2) after one minute, upper arm cuff was inflated to seven mmHg below diastolic blood pressure and arterial inflow recorded for 30 seconds; (3) forearm venous capacitance was measured after maintaining the upper arm cuff pressure for an additional five minutes; and (4) venous outflow was assessed following the release of the upper arm blood pressure cuff.

#### Control

After a 10 minute period of supine rest the upper arm cuff was inflated to a pressure of 240 mmHg for five minutes. RHBF was measured upon release of the cuff pressure back to a pressure seven mmHg below diastolic blood pressure. Venous capacitance and venous outflow were assessed following the five minute venous occlusion period.

#### Pre-elevation

After an additional 10 minute rest period the arm of the participant was passively raised by an investigator to 90° above heart level for two minutes. Following the two minute *Pre-elevation *period the upper arm cuff was again inflated to 240 mmHg before the forearm was repositioned as described above for five minutes. RHBF, venous capacitance and venous outflow were then measured as described for Condition 1.

#### Order Effect

Initially, 21 subjects were tested using the rest, Control, and Pre-elevation testing sequence. Given the initial data suggested a possible order effect; an additional 14 subjects were tested with Pre-elevation preceding the Control condition.

### Autonomic Nervous Function Assessments

Heart rate variability (HRV) was used to assess autonomic nervous function. ECG electrodes were applied on the participant's chest and attached to the Biopac MP 100. Data were recorded continuously via software program Acqknowledge (model MP100A, Biopac Inc., Santa Barbara, CA). ECG data collected before and during the two minute arm elevation were analyzed for mean heart period (mean R-R interval), standard deviation. HRV analyses were made in accordance with the recommendations of the Task Force of the European Society of Cardiology and the North American Society of Pacing and Electrophysiology [9].

### Data Analysis

The vascular function indices were analyzed as described previously [10]. Briefly, arterial inflow was recorded at a paper speed of five mm·sec^-1 ^and values were derived from the slope drawn at a best-fit tangent using the first three pulses. Values were calculated with the formula, 60 seconds multiplied by the full chart range and divided by the longitudinal distance (mm), which reflects the slope between the baseline to the top of the recording paper. RHBF following the conditions were calculated by dividing the product of paper speed (25 mm·sec^-1^) and time (60 seconds) by the horizontal distance (mm) needed for the volume slope to increase by 20 mm vertically. Forearm venous capacitance was measured as the vertical distance (mm) representing the increase in the forearm volume graph after the designated period (as described for each experiment) for venous filling. Finally, forearm venous outflow was derived from a tangent line that represents the vertical drop in the volume graph from the excursion line and drawn at 0.5 and two seconds after the release of the venous occlusion pressure [10].

### Statistical Analysis

Statistical analyses were performed using SPSS for Windows (*Version 11.0*). The data are presented as the mean ± SD. To examine differences for arterial inflow, and vascular capacitance, and venous outflow (at rest and following the Control and Pre-elevation conditions) a repeated measure design, with subsequent pairwise comparisons was used. In addition, multivariate analysis of variance was used to determine the difference in hemodynamic responses (heart rate and blood pressure) at rest and following the Control and Pre-elevation conditions. Pearson correlation was used to examine the relationships between the vascular measures. Alpha was set a priori at 0.05.

## Results

### Participants' Characteristics

Thirty-five individuals (16 males, 19 females) between the ages of 19–27 participated in this study. Participant characteristics are presented in Table 1.

**Table 1:** Participant Characteristics

Characteristics	Mean ± SD
Age (y)	22 ± 2
Height (cm)	170 ± 10
Weight (kg)	68.77 ± 14.43
Forearm Circumference (cm)	24 ± 3

Values are mean ± SD

### Resting Blood Flow Measures

The average resting arterial inflow for the group was 2.42 ± 1.11 ml·100 ml^-1^·min^-1^, and average venous capacitance and outflow were 2.90 ± 0.90% and 34.82 ± 10.33 ml·100 ml^-1^·min^-1^, respectively.

### Control

The average RHBF measures following five minutes of upper arm occlusion was 19.33 ± 4.50 ml·100 ml^-1^·min^-1^. Venous capacitance after occlusion was significantly lower compared to rest (*p *< 0.05). Venous outflow after occlusion was similar compared to the resting measure.

### Pre-elevation

**Figure 1:**
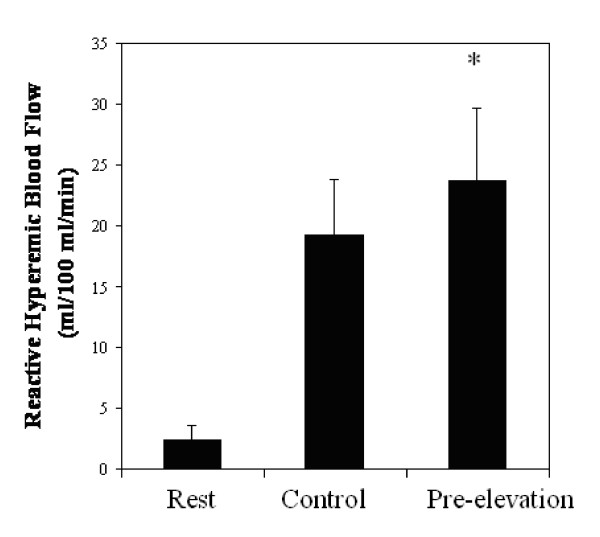
**Blood Flow Responses at Rest and following 5-min of Occlusion with (Pre-Elevation) and without (Control) arm elevation**. Values are mean ± SD, * = Significantly different from Rest and Control

**Figure 2:**
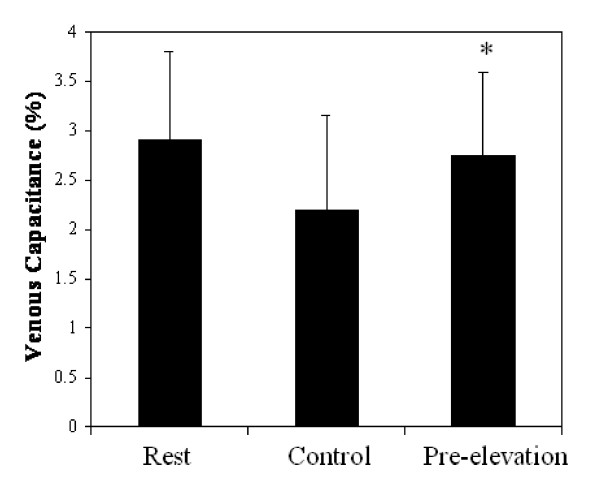
**Venous Capacitance Measures at Rest and following 5-min of Occlusion with (Pre-Elevation) and without (Control) arm elevation**. Values are mean ± SD, * = Significantly different from Control

**Figure 3:**
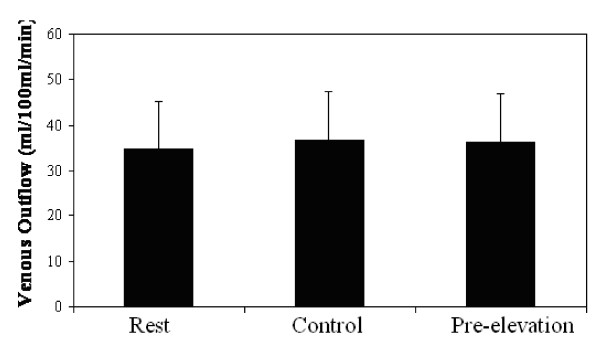
**Venous Outflow Measures at Rest and following 5-min of Occlusion with (Pre-Elevation) and without (Control) arm elevation**. Values are mean ± SD

The average RHBF measures following five minutes of upper arm occlusion, preceded by two minutes of arm elevation was 23.76 ± 5.95 ml·100 ml^-1^·min^-1 ^and significantly greater (30%) compared to the Control (see Figure 1). Venous capacitance was also significantly higher compared to the Control (see Figure 2). However, venous outflow was similar between the two conditions (see Figure 3).

### Comparison of Conditions

The repeated measure design revealed a significant main effect for arterial inflow and venous capacitance, but not for the venous outflow measures. Pairwise comparisons revealed a significant difference between all conditions for arterial inflow (rest, Control and Pre-elevation conditions), and between the Control and Pre-elevation conditions for venous capacitance.

### Heart Rate/Blood Pressure/R-R Interval

Average values for heart rate and blood pressures are shown in Table 2. There were no significant changes in these measures from rest to Control and Pre-elevation. In addition, the R-R intervals before (0.88 ± 0.35 sec) and during arm elevation (0.9 ± 0.35 sec) did not reveal significant differences.

**Table 2:** Heart Rate and Blood Pressure Responses

	Rest	Control	Pre-Elevation
HR (bpm)	62 ± 10	61.5 ± 10.1	58.8 ± 10.0
SBP (mmHg)	115 ± 10	107 ± 24.8	111 ± 10.49
DBP (mmHg)	64 ± 7	63 ± 7.7	62.5 ± 8.2

Values are mean ± SD HR: Heart rate, SBP: Systolic blood pressure, DBP: Diastolic blood pressure

Pearson product moment correlations for between the vascular measures, blood pressure and mean R-R intervals are presented in Table 3. Significant associations were noted between RHBF and the venous measures, as well as diastolic blood pressure. In addition, the average mean R-R intervals were significantly associated with both systolic and diastolic blood pressure, but not with the vascular measures.

**Table 3:** Pearson Product Moment Correlation for Hemodynamic Measures

	RHBF	Venous capacitance	Venous outflow	SBP	DBP	R-R
RHBF (ml·100 ml^-1^·min^-1^)	1					
Venous capacitance (% volume change)	.407*	1				
Venous outflow (ml·100 ml^-1^·min^-1^)	.347*	.826**	1			
SBP (mmHg)	.266	.126	.080	1		
DBP (mmHg)	.329*	.389*	.210	.506**	1	
R-R (s)	.288	.261	.151	.562**	.501*	1

RHBF: Reactive hyperemic blood flow following arm elevation, SBP: Systolic blood pressure following arm elevation, DBP: Diastolic blood pressure following arm elevation, R-R: heart rate intervals at rest.

** Correlation is significant at the 0.01 level (2-tailed).

* Correlation is significant at the 0.05 level (2-tailed).

## Discussion

The main objective of this study was to examine the effect of adding a two minute period of passive arm elevation prior to five minutes of upper arm occlusion on the RHBF response. The maneuver is thought to contribute to an acute reduction in venous volume in the arm, without a significant change in heart rate, blood pressure, and measures of heart rate variability. The present study indicates the maneuver contributes to a 23% greater RHBF response compared to five minutes of arterial occlusion without venous emptying. This finding suggests the initial volume in the venous system influences the RHBF responses in the forearm.

The values obtained for resting and RHBF without prior arm elevation are similar to other published reports from our laboratory [2,3], and quite similar to a low cardiorespiratory fitness group previously studied [11]. The underlying assumption of the model is that the period of occlusion renders the tissue ischemic and, perhaps, hypoxic. Upon release of the occluding pressure, the tissue is rapidly reperfused and the magnitude of the response is dependent on several mechanisms, such as metabolic waste products that accumulate during the occlusion and stimulate the opening of many precapillary sphincters in the tissue beds [1]. In addition, myogenic and neural reflexes are also thought to play an important role in the phenomenon [8]. Overall the response evaluates the autoregulatory ability of the tissue to respond to a stressor.

A unique finding of the present study was that venous emptying prior to forearm occlusion resulted in significantly greater RHBF responses. These differences were noted regardless of the order of testing. Importantly, other hemodynamic measurements, including blood pressure and mean R-R intervals were similar during both conditions, suggesting that central factors did not influence the observed differences. Although the current study was not designed to explain the change in the RHBF response under the two conditions, at least two mechanisms could have contributed to the observed effect. These mechanisms include a reduction in local arterial and venous pressure and an alteration in the venoarterial reflex.

In regard to the first mechanism, the pressure gradient across the tissue bed may have been sufficiently altered after the venous emptying maneuver. Consequently, the driving pressure across the tissue bed would have been greater, and contributed to the higher RHBF response. The finding in the present study is quite consistent with the observation by Tschakovsky and Hughson [8]. These investigators observed a significantly greater blood flow through a single conduit artery after arm elevation.

A second contributor to the increased RHBF following venous emptying may be alterations in a local reflex control system known as the venoarterial reflex [8]. The venoarterial reflex is a local axon reflex that responds to stretching of the venous wall and contributes to arterial vasoconstriction [12]. Some researchers [13,14] suggest that a reduction of the venous pressure may result in the withdrawal of venoarterial reflex and contribute to greater arterial vasodilation.

In addition to the proposed mechanisms discussed above, it is also possible that the venous emptying maneuver may elicit a greater hyperemic stimulus, secondary to a greater drop in oxygen tension in the region under study. Evidence for this stems from studies in which positional changes in the lower limbs of patients resulted in a drop in regional perfusion indices [15]. We were incapable to determine the extent of a change in oxygen tension during the conditions imposed.

With regard to venous function, findings from the present investigation reveal a 25% reduction in venous capacitance after arm occlusion compared to venous capacitance at rest. The explanation for this observation is not clear, but may involve a greater number of deep tissue capillary beds that are opened following the period of occlusion. This physiologic alteration may contribute to less expansion of the arm and stretching of the strain gauge. Interestingly, the present study showed that venous capacitance after the two minute arm elevation followed by occlusion showed similar venous capacitance values as observed at rest. We are unsure as to how a decrease in forearm venous volume prior to these measures could have contributed to this change.

In contrast, measures of venous outflow were not different between the conditions. This lack of difference may be quite important in that it provides indirect evidence that the venous outflow is determined by the barriers within the venous system rather than by the volume in the veins at the time of the measurement. Therefore this measure may in fact be independent of the volume, and provide a better reflection of the impedance within the venous system. Clearly a better understanding of this phenomenon must come through subsequent work in this area.

It is interesting to consider the significant association between venous outflow measures and RHBF, possible indicating an interplay between the systems. The relationship indicates that those who have greater arterial inflow also have greater venous outflow. Hester et al. [16] hypothesized that vasoactive substances may have a role in this interplay. During an increasing metabolic demand, as in exercise, the close venular-arteriolar pairing allows for diffusion of vasoactive substances from the venular blood to the arterioles. Red blood cells release adenosine triphosphate, which may stimulate the venular endothelium to release vasoactive metabolites of arachidonic acid [16]. Consequently, Hester also reported that increased tissue adenosine results in upstream arteriolar dilation through venular-arteriolar diffusion [17]. Injections of neoroephinephrine into precapillary vessels are the other evidence of venular controls of the arterioles, and it results in upstream arteriolar constriction at a point where the venule parallels the arteriole [18].

Furthermore, it is interesting to consider the influence of conditions that may result in venous congestion, on arterial inflow characteristics and perhaps performance. Zelis and colleagues [4], who examined vascular function in patients with congestive heart failure, identified that the patient group had significantly higher venous pressures, and lower resting (49%) and peak RHBF (25%) compared to healthy controls. Given the clear evidence that the cardinal symptom of patients with heart failure, and others who suffer from venous congestion [19,20] is exercise intolerance, future studies are needed to determine if the problems are in part due to a reduction in the pressure gradient across the muscle bed. In light of previous work showing that a greater perfusion gradient across the tissue beds during muscular contractions contributes to a delay in the onset of fatigue [21], one might speculate that a lower perfusion gradient across the tissue bed may result in a slower removal of metabolites, thereby contributing to the early onset of fatigue and exercise intolerance.

Obviously, the interpretation of these data must be guarded in terms of the limitations of the study. Plethysmography is an indirect method to measure vascular functions and blood flow. The changes in circumferences as measured by the strain gauge do not allow individual assessment of fluid changes within specific compartments. Arterial, venous and interstitial areas cannot be assessed independently. However, a high correlation (r^2 ^= 0.87–0.98) between venous occlusion plethysmography and Doppler ultrasound of the brachial artery suggest the change does reflect blood inflow [1,22].

Finally, the present findings may have potential methodological and physiological implications. In terms of the methodological implication one must consider the importance of emptying the venous system if indeed the RHBF response following venous emptying is greater than the traditional methods. In terms of the physiological implication, the potential influence of the venous system on arterial inflow may broaden our understanding of the interplay between the two parts of the circulation, and may widen our strategies to include efforts to modify both systems.

## Conclusion

In conclusion, the present study suggests that venous emptying prior to arterial occlusion may contribute to a 23% greater RHBF response as compared to occlusion without prior emptying. The present finding has potential methodological and physiological consequences. In terms of the methodological importance one must consider the importance of emptying the venous system prior to measuring RHBF. Moreover, it is important to consider the possible influence of certain interventions, such as diet, exercise, and pharmacology, on venous volume measures. Knowledge of the influence of interventions on venous measures will subsequently provide a better understanding of the interplay between the arterial and venous systems.

## Authors' contributions

ZB: participated, coordinated and executed the study design, and drafted the manuscript.

ET: assisted in the coordination and execution of the study design.

AAAE: participated, and assisted in the coordination of the study design.

DAD: assisted in the coordination and execution of the study design.

MAW: conceived of the study, and participated in its design and coordination and helped to draft the manuscript. All authors read and approved the final manuscript.
